# Enhancing therapeutic efficacy in luminal androgen receptor triple-negative breast cancer: exploring chidamide and enzalutamide as a promising combination strategy

**DOI:** 10.1186/s12935-024-03313-5

**Published:** 2024-04-09

**Authors:** Ya-Xin Zhao, Han Wang, Si-Wei Zhang, Wei-Xin Zhang, Yi-Zhou Jiang, Zhi-Ming Shao

**Affiliations:** 1 Key Laboratory of Breast Cancer in Shanghai, Department of Breast Surgery, Fudan University Shanghai Cancer Center, No. 270 Dong’an Road, Shanghai, 200032 People’s Republic of China; 2 Department of Oncology, Shanghai Medical College, Fudan University, Shanghai, 200032 People’s Republic of China

**Keywords:** Triple-negative breast cancer, Luminal androgen receptor, HDAC inhibitors, Combination therapy, Synergistic effect

## Abstract

Extensive exploration of the molecular subtypes of triple-negative breast cancer (TNBC) is critical for advancing precision medicine. Notably, the luminal androgen receptor (LAR) subtype has attracted attention for targeted treatment combining androgen receptor antagonists and CDK4/6 inhibitors. Unfortunately, this strategy has proven to be of limited efficacy, highlighting the need for further optimization. Using our center’s comprehensive multiomics dataset (n = 465), we identified novel therapeutic targets and evaluated their efficacy through multiple models, including in vitro LAR cell lines, in vivo cell-derived allograft models and ex vivo patient-derived organoids. Moreover, we conducted flow cytometry and RNA-seq analysis to unveil potential mechanisms underlying the regulation of tumor progression by these therapeutic strategies. LAR breast cancer cells exhibited sensitivity to chidamide and enzalutamide individually, with a drug combination assay revealing their synergistic effect. Crucially, this synergistic effect was verified through in vivo allograft models and patient-derived organoids. Furthermore, transcriptomic analysis demonstrated that the combination therapeutic strategy could inhibit tumor progression by regulating metabolism and autophagy. This study confirmed that the combination of histone deacetylase (HDAC) inhibitors and androgen receptor (AR) antagonists possessed greater therapeutic efficacy than monotherapy in LAR TNBC. This finding significantly bolsters the theoretical basis for the clinical translation of this combination therapy and provides an innovative strategy for the targeted treatment of LAR TNBC.

**Supplementary Information:**

The online version contains supplementary material available at 10.1186/s12935-024-03313-5.

## Background

Breast cancer has emerged as one of the most common malignant tumors globally, posing a grave threat to women’s health and lives [1, 2]. Triple-negative breast cancer (TNBC) is characterized by the absence of estrogen receptor (ER), progesterone receptor (PR), and amplification of human epidermal growth factor receptor 2 (HER2) [3]. Due to the lack of effective treatment targets, chemotherapy remains the primary course of TNBC treatment; however, the efficacy and side effects need improvement, representing a major challenge in breast cancer treatment [3, 4]. The emergence of molecular subtyping of TNBC has advanced the search for therapeutic targets and clinical trials [3, 5–7]. Notably, the luminal androgen receptor (LAR) subtype has high expression of androgen receptor (AR), a higher proportion in the Asian population, and a relatively poor prognosis. Unfortunately, previous treatment efficacy based on the combination of anti-androgen receptor drugs and CDK4/6 inhibitors was unsatisfactory, and therefore, the treatment strategy urgently needs optimization [7].

Histone deacetylases (HDACs) are crucial in chromatin remodeling and play a pivotal role in the epigenetic regulation of gene expression [8]. HDAC inhibitors (HDACis) can target the acetylation of histones and regulate various biological processes, such as cell apoptosis, immune modulation, and tumor angiogenesis [9–11]. As such, HDACs have emerged as important targets for cancer therapy. Currently, four HDACis has been approved by the Food and Drug Administration (FDA), namely chidamide, vorinostat, romidepsin, and panobinostat, which are effective in treating T-cell lymphoma and multiple myeloma [12]. However, their efficacy in solid tumors is limited due to pharmacokinetic reasons, and combination therapy is expected to improve this situation. In recent studies, HDACis have been successfully used in combination with various agents such as DNA-damaging agents, taxanes, death receptor agonists, and hormone therapies [13]. The FDA has approved three AR antagonists, enzalutamide, apalutamide, and darolutamide, for treating nonmetastatic castration-resistant prostate cancer [14]. Combining HDACis and AR antagonists has proven to be effective in several studies on prostate cancer [15, 16]. In breast cancer cases, HDACis can inhibit metastasis and growth through the IL - 6/STAT3 signaling pathway, while AR antagonists can enhance the antitumor effects of PARP inhibitors in AR-positive breast cancer by regulating the DNA damage response [17, 18]. Therefore, the combination therapy of HDACis and AR antagonists may be a possibility for treating breast cancer, especially LAR-type TNBC.

Chidamide is the first subtype-selective HDAC inhibitor synthesized and developed independently in China. It is the only HDACi approved by the National Medical Products Administration (NMPA) for clinical trials and is primarily used to treat relapsed or refractory peripheral T-cell lymphoma [19]. Studies have shown that chidamide can regulate various malignant biological behaviors of tumor cells, including inhibiting cell proliferation and migration, inducing apoptosis, and enhancing the efficacy of chemotherapy drugs or immune checkpoint inhibitors. These findings suggest that combination therapy with chidamide and other drugs holds great potential for effective cancer treatment [20–24]. In recent clinical studies, the combination of chidamide and endocrine therapy has shown significant therapeutic effects on advanced hormone receptor-positive breast cancer [25, 26]. Therefore, further exploration of the combination of HDACis with anti-androgen endocrine therapy for LAR-type TNBC is highly significant and promising.

In this study, we examined the sensitivity of LAR cell lines to chidamide and enzalutamide and verified the synergistic effect of the combination of chidamide and enzalutamide by a drug combination assay. The synergistic effect of combination therapy was verified in vivo using allograft models. Furthermore, we found that combination therapy may inhibit tumor proliferation by regulating metabolism and autophagy. Our results verified that the combination of HDACis and AR antagonists has greater therapeutic efficacy in LAR-type TNBC, demonstrated a theoretical basis for the clinical translation of combination therapy and provided a new idea for the targeted treatment of LAR-type TNBC patients.

## Methods

### Patient cohorts

Our study included one multiomics cohort of patients with TNBC breast cancer who underwent surgery and adjuvant chemotherapy at Fudan University Shanghai Cancer Center (FUSCC). This cohort consisted of 465 patients, of whom 360 had RNA-seq data (n = 81 for the LAR subtype; n = 279 for the non-LAR subtype). Further details about this cohort have been described in our previous study [6].

### Human and mouse cell lines

The human breast cancer cell lines MDA-MB-453 and CAL-148 were obtained from American Type Culture Collection (ATCC) and Guohong Hu’s laboratory (Shanghai Institute of Nutrition and Health, University of Chinese Academy of Sciences, Shanghai, China), respectively. The TS/A mouse TNBC cell line was obtained from Yibin Kang’s laboratory (Princeton University, USA). All cell lines were classified into the TNBC LAR subtype, as previously described [27]. Cell viability, mycoplasma contamination and short tandem repeat analysis were monitored to identify cell lines. MDA-MB-453, CAL-148 and TS/A cells were maintained in high-glucose DMEM (Basal Media, #L110) supplemented with 10% FBS and 1% penicillin–streptomycin at 37 ℃ in a 5% CO_2_ incubator.

### Transplantation models

Five- to six-week-old female BALB/c mice and nude BALB/c mice were obtained from Shanghai Jihui Laboratory Animal Care. To establish allograft models,  5 × 10^5 ^TS/A mouse breast cancer cells were injected into the mammary fat pad region of each BALB/c mouse. Mice were housed in a specific pathogen-free facility with individually ventilated cages, under 12 h light/dark cycles and at an ambient temperature of 20–22 ℃ and humidity of 60 ± 10%. They were provided with free access to a standard rodent diet (Jiangsu Xietong Pharmaceutical Bio-engineering, 1010013) and water ad libitum.

### Organoid culture

Human breast cancer organoids were stored in a biobank following a previously published protocol [28]. The organoids were resuscitated from the biobank and resuspended in BME type-2 (Trevigen, 3533-010-02). Then, the suspension was plated in a 300 μl drop within a 12 mm, 0.4 μm inner transwell chamber (Corning). The drop was solidified by a 30-min incubation at 37 ℃ and 5% CO_2_ with 1 ml of breast cancer organoid medium (Advanced DMEM/F12 supplemented with R-spondin-1 [500 ng/ml, PeproTech], Noggin [100 ng/ml, PeproTech], Neuregulin [5 nM, PeproTech], Estradiol [5 nM, Sigma], HEPES [1 mM, Gibco], GlutaMAX [1X, Gibco], Nicotinamide [5 mM, Sigma], N-Acetylcysteine [1.25 mM, Sigma], B-27[1X, Gibco]), A83-01 [0.5 mM, Tocris], Primocin [1X, InvivoGen], SB-202190 [500 nM, Selleck], Y27632 [5 mM, Selleck], FGF10 [20 ng/ml, PeproTech], FGF7 [5 ng/ml, PeproTech] and EGF [5 ng/mL, PeproTech]).

### In vitro cell viability assays

To conduct cell viability assays, cells were plated at optimal seeding densities in 96-well plates and allowed to adhere overnight. The optimal seeding densities were established based on each cell line reaching 75–80% confluence at the end of the assay. The day after the cells reached confluence, the growth medium was removed, and 100 μl of fresh medium containing various inhibitors at corresponding concentrations was added to each well. After 72 h, cell viability was assessed by Cell Counting Kit-8 (Yeasen, #40203ES92). The absorbance was measured at 450 nm (A450). The concentration of the drug resulting in 50% inhibition of cell viability (IC50) was calculated using four-parameter logistic curve fitting. Additionally, CompuSyn was leveraged to calculate the combination index (CI).

### In vivo mouse studies

All animal experiments were conducted in accordance with protocols approved by the Research Ethical Committee of Fudan University Shanghai Cancer Center. The in vivo experimental protocols were all reviewed and approved by the Institutional Animal Care and Use Committee. Mice with transplanted tumors were randomly divided into 4 groups: (1) treated with 0.2% carboxymethyl cellulose and 0.1% Tween 80; (2) treated with chidamide (20 mg kg^−1^, oral gavage daily); (3) treated with enzalutamide (5 mg kg^−1^, oral gavage daily); and (4) treated with chidamide and enzalutamide. Tumor size was measured daily. Tumor volume in mm^3^ was calculated using the following formula: tumor volume = 0.5 × L × W^2^, where L is the longest dimension and W is the perpendicular dimension.

### Flow cytometry analysis

After in vivo experiments, mouse tumors were rapidly excised and mechanically dissociated in PBS using scissors. The tumors were then digested in serum-free RPMI supplemented with 20 mg/ml DNase I (Roche), 20 mg/ml Dispase II (Roche) and 20 mg/ml collagenase I (Sigma) for 30–60 min at 37 °C with rotation to promote dissociation. The resulting single-cell suspensions were passed through 70 μM strainers twice, and red blood cells in the tumor samples were lysed with red blood cell lysis buffer (eBioscience, #00-4333-57) for 5 min at room temperature. Then, the single-cell suspensions were washed in Cell Staining Buffer (BioLegend) and incubated with the indicated flow antibodies at 4 °C for 30 min. Prior to staining with antibody panels, cells were blocked with a monoclonal antibody against CD16/32 (BioLegend) for 15 min at 4 °C. All the antibodies and reagents used for flow cytometry included ZombieRED (ECD, Biolegend, #423110), CD45 (clone 30-F11, Biolegend, #103116), CD3e (clone 145-2C11, Biolegend, #100328), CD4 (clone RM4-5, Biolegend, #100536), CD8 (clone 53–6.7, Biolegend, #100706), GZMB (clone QA16A02, Biolegend, #372208), PRF1 (clone S16009B, Biolegend, #154404), F4/80 (clone BM8, Biolegend, #123127), CD11b (clone M1/70, Biolegend, #101206), CD86 (clone GL-1, Biolegend, #105006) and CD206 (clone C068C2, Biolegend, #141719).

### RNA-seq

Tumors collected from in vivo experiments were treated with TRIzol reagent to isolate total RNA. Library construction was performed with the generated 150 bp paired-end reads, and RNA-seq data sequence analysis was carried out on the Illumina HiSeq platform. Pheatmap, Kmeans and DESeq2 R packages were used for differential gene cluster analysis. Finally, GO and KEGG enrichment analyses were performed using the clusterProfiler and GSVA packages. CIBERSORT calculated with the ‘‘kappa’’ function in R was used to calculate the abundance of 22 types of immune cell subsets in each sample. ESTIMATE (Estimation of STromal and Immune cells in MAlignant Tumor tissues using Expression data) was used to rank tumor and stromal scores. A gene coexpression network was constructed by the WGCNA package and the demonstration of module feature vector clustering involves assigning different colors as labels. The ConsensusClusterPlus package was used to define clusters in different groups. Survival and glmnet packages were used to perform univariate and multivariate Cox regression and Lasso-Cox regression analyses.

### Quantification and statistical analysis

Statistical significance was determined using an unpaired Student’s t test, Mann–Whitney test, chi-square test or Kruskal−Wallis test when appropriate. All data are presented as the mean ± SEM of at least three independent experiments unless otherwise indicated in the figure legend. All cell-based in vitro experiments were independently repeated three times in triplicate. Two-sided p values less than 0.05 were considered statistically significant. The p values and sample size can be found in the main and supplementary figure legends. All figures were created by R software (http://www.R-project.org, version 3.5.2) and Sangerbox Tools [29]. All statistical analyses were performed with either R software or GraphPad Prism software (version 9.0).

## Results

### Patient cohorts and study design

We utilized the FUSCC dataset, consisting of 465 cases, 360 of which had transcriptomic data, 279 samples with whole-exome sequencing (WES) results, and 401 samples with somatic copy-number alteration (SCNA). In this cohort, we analyzed and compared the clinical and pathological characteristics between LAR subtype and non-LAR subtype patients. Our findings revealed significant differences between the two subtypes in terms of age at onset, menopausal status, histological grade, Ki67 proliferation index and lymph node status (Table 1). Notably, LAR TNBCs exhibited higher histological grade, lower Ki67 proliferation index and a higher frequency of lymph node metastasis.

**Table 1:** Clinicopathological characteristic of LAR vs non-LAR patients in the FUSCC cohort

Characteristic	Group		P value
	LAR (n = 83)	non-LAR (n = 303)	
Age (years)			< 0.001
≤ 50	17 (20.5%)	146 (48.2%)	
> 50	66 (79.5%)	157 (51.8%)	
Size (cm)			0.794
≤ 2	30 (36.1%)	111 (36.6%)	
> 2	53 (63.9%)	191 (63%)	
Unknown	0	1 (0.3%)	
Menopause			0.004
Yes	64 (77.1%)	175 (57.8%)	
No	19 (22.9%)	123 (40.6%)	
Unknown	0	5 (1.7%)	
Grade			< 0.001
≤ 2	28 (33.7%)	38 (12.5%)	
> 2	51 (61.4%)	230 (75.9%)	
Unknown	4 (4.8%)	35 (11.6%)	
Ki67 (%)			< 0.001
< 30	30 (36.1%)	43 (14.2%)	
≥ 30	46 (55.4%)	257 (84.8%)	
Unknown	7 (8.4%)	3 (1.0%)	
LN status			0.008
Positive	44 (53.0%)	108 (35.6%)	
Negative	38 (45.8%)	194 (64.0%)	
Unknown	1 (1.2%)	1 (0.3%)	

*Chi-square tests were used to analyze clinicopathological varibles, with patients of unknown information duly excluded

**Fig. 1:**
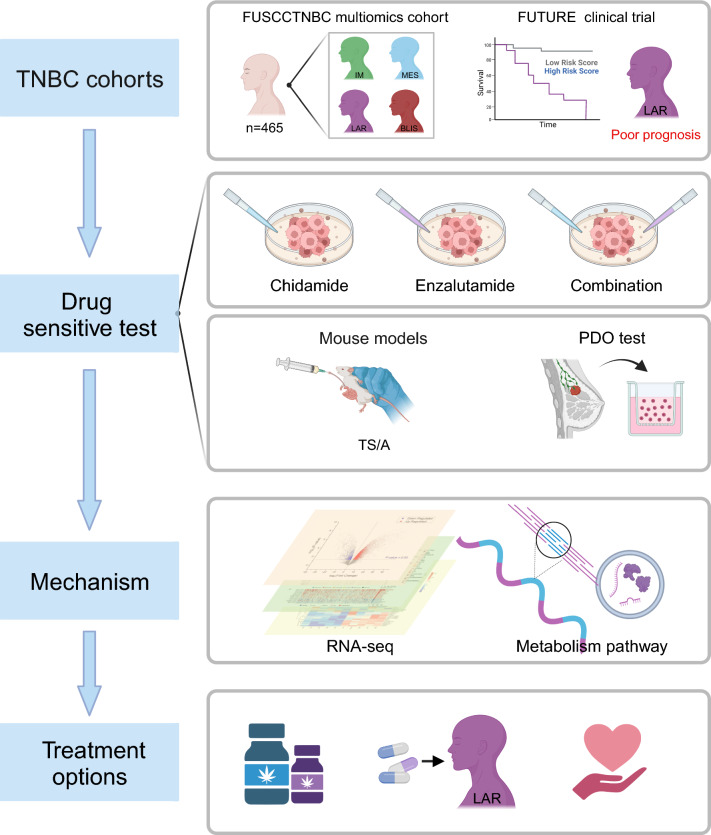
Workflow of the analytical process conducted in this study. The analytical process performed in this study followed a structured framework: This study utilized multi-omics cohort from FUSCC to identify potential therapeutic targets for the LAR subtype. The efficacy of treatment strategies and potential molecular mechanisms were investigated through in vivo in vitro ex vivo drug sensitivity experiments, as well as RNA sequencing. The aim of this study was to propose potential clinical treatment strategies specifically for the LAR subtype. LAR luminal androgen receptor, IM immunomodulatory, BLIS basal-like and immune-suppressed, MES mesenchymal-like, FUSCC Fudan University Shanghai Cancer Center

Based on different clinical and molecular characteristics, a specific targeted therapeutic strategy was proposed for the LAR subtype without ERBB2 mutation in the FUTURE clinical trial (anti-AR plus anti−CDK4/6). However, the trial results showed that only one of eight assessable patients presented with stable disease (SD), while the remaining seven patients displayed progressive disease (PD) [7]. This finding suggests that combining anti-AR therapy with other targeted therapies might be more appropriate. Therefore, in this study, we conducted a comprehensive analysis of multiomics data within the FUSCC TNBC cohorts. Then, we performed corresponding in vitro and in vivo experiments to explore other potential therapeutic strategies, as well as the underlying mechanisms (Fig. 1).

### Patients with LAR TNBC are subject to epigenetic regulation

**Fig. 2:**
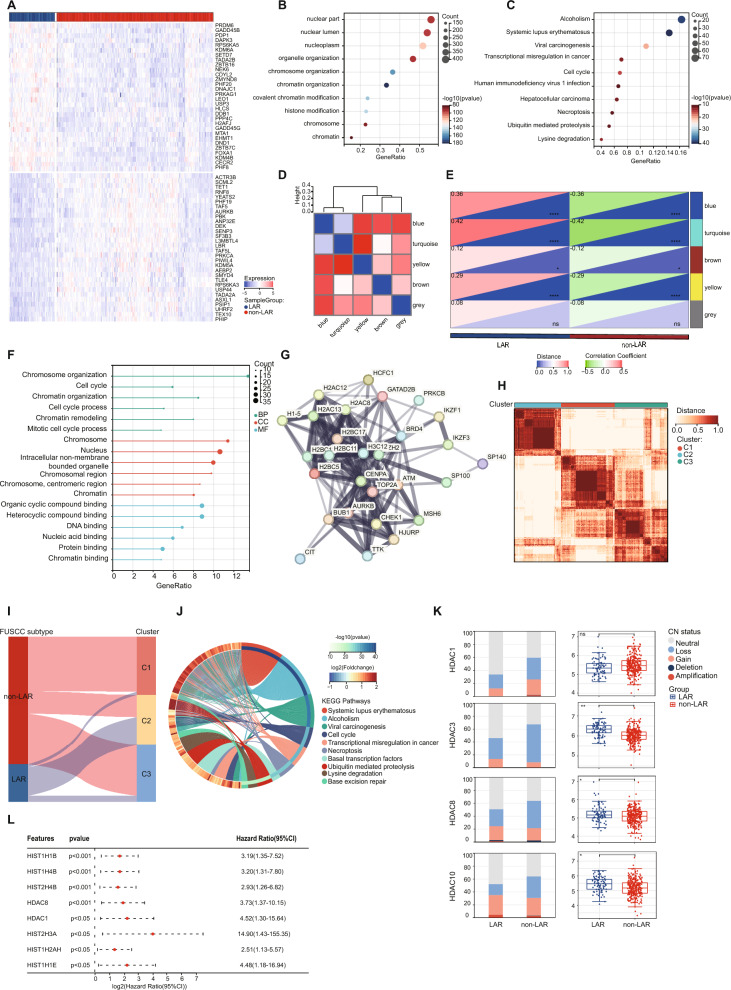
Patients with LAR TNBC are subject to epigenetic regulation. **A** Heatmap showing the top 30 differentially expressed epigenetic-related genes (ERGs) filtered by P  <  0.05 and log_2_FC > 1. **B–C** Enrichment of ERGs in signaling pathways as determined by GO (**B**) and KEGG analysis (**C**). **D–E** Construction of ERG WGCNA network. The demonstration of module feature vector clustering (**D**). Correlation analysis between each module and TNBC subtype feature (**E**). Color labels are exclusively employed to differentiate between various gene modules and hold no intrinsic significance. **F** Functional annotation of hub genes in the LAR subtype processed by GO enrichment analysis. **G** PPI network showing coexpressed proteins of WCGNA hub genes. **H** ConsensusCluster determined by ERGs in the FUSCC TNBC cohort. **I** The Sankey diagram displaying relationships among TNBC subtypes and gene clusters. **J** Chord plot demonstrating the top 10 enriched signaling pathways. **K** Copy number alterations (left) and transcriptional expression levels (right) of representative HDAC genes. **L** Univariate Cox regression analysis of ERGs in the LAR subtype of TNBC. Abbreviations: LAR, luminal androgen receptor; TNBC: Triple-Negative Breast Cancer; WGCNA: Weighted Gene Co-expression Network Analysis; ERGs: Epigenetic-Related Genes; HDAC: Histone Deacetylases. Statistical analysis was performed by the Student’s t test (E, L, K) or Mann–Whitney test (B, C, J). ****Indicates P < 0.0001; ***indicates P < 0.001; **indicates P < 0.01; *indicates P < 0.05; ns indicates no significance

Initially, we performed differential gene analysis using epigenetic-related genes (ERGs) obtained from the EpiFactors database [30]. We found significant differences between LAR and non-LAR subtype patients (Fig. 2A). Gene Ontology (GO) enrichment analysis also highlighted chromosome organization and histone modification as prominent biological processes associated with the LAR subtype (Fig. 2B). Additionally, KEGG (Kyoto Encyclopedia of Genes and Genomes) pathway analysis demonstrated a greater connection between the LAR subtype and epigenetic-related pathways such as transcriptional misregulation, cell cycle, and human immunodeficiency (Fig. 2C). Then, we developed a weighted gene coexpression network analysis (WGCNA) to identify a coexpression model for the ERGs in the LAR subtype (Fig. 2D,E). Next, we conducted GO and KEGG analyses on the hub genes. Likewise, this analysis revealed enrichment of epigenetic-related pathways, such as cell cycle, chromatin organization and chromatin remodeling processes, within the LAR subtype (Fig. 2F). A PPI (Protein–Protein Interaction) network was constructed to explore the coexpression proteins. Through functional enrichment analysis, we found that this network was mainly associated with histone kinase activity and the cell cycle, which was consistent with our previous results (Fig. 2G).

To further verify these findings, we employed ConsensusCluster to stratify the FUSCC TNBC dataset with epigenetic-related genes, resulting in the identification of three clusters: cluster 1 (n = 128), cluster 2 (n = 109), and cluster 3 (n = 123). Notably, a majority of LAR-subtype patients were categorized under cluster 2 (Fig. 2H, I). This cluster was enriched with the cell cycle, transcriptional misregulation in cancer, and the necroptosis pathways, which is consistent with the enriched pathways in the LAR subtype (Fig. 2J).

Next, our focus shifted toward exploring key epigenetic-related regulators in the LAR subtype, which might be potential drivers and therapeutic targets. As previously described, HDACs play a vital role in epigenetic regulation [8]. Interestingly, the LAR subtype of TNBC exhibited a lower frequency of loss/deletion or a higher frequency of gain/amplification in several HDACs such as HDAC1, HDAC3, HDAC8, and HDAC10 (Fig. 2K). Consistently, LAR TNBCs had elevated transcriptional levels of most of the aforementioned HDACs. Moreover, using univariate Cox regression analysis, histone-related genes, including HDACs, predominantly predicted a higher risk of recurrence in the LAR subtype (Fig. 2L). This suggests that HDACs could serve as promising targets for LAR TNBCs.

Taken together, epigenetic regulation has a greater significance in the LAR subtype of TNBC, particularly in the context of histone acetylation and deacetylation. Therefore, LAR TNBCs may have a higher potential for benefiting from therapeutic interventions targeting epigenetic regulators, such as HDACs.

### Synergistic effect of chidamide combined with enzalutamide in vitro

In this study, we chose chidamide, a subtype-selective HDAC inhibitor of HDAC 1/2/3/10 [31]. As a candidate drug compatible with the anti-AR agent enzalutamide for treating LAR TNBC. It is worth noting that chemotherapy is the main strategy for TNBC, and LAR TNBCs are characterized by a high frequency of PI3K mutations [6]. Therefore, we selected the PI3Ki alpelisib and paclitaxel as controls.

**Fig. 3:**
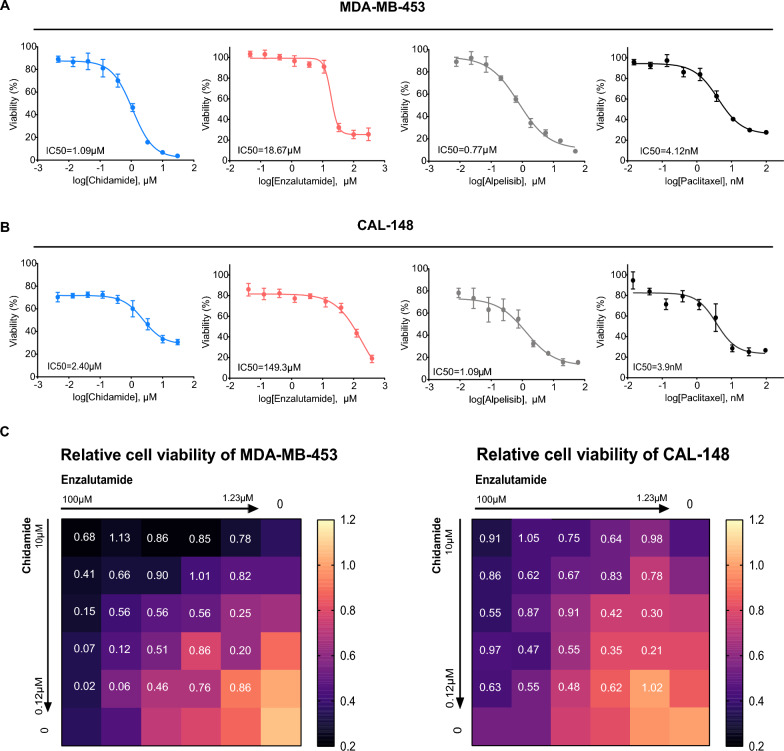
Synergistic effect of chidamide combined with enzalutamide in vitro. **A, B** Cell viability of MDA-MB-453 and CAL-148 cell lines treated with chidamide or enzalutamide. The IC50 values are shown in the left corner of each figure. **C** Heatmap showing the cell viability and CI value of chidamide and enzalutamide in MDA-MB-453 and CAL-148 cell lines at different drug concentrations

We conducted growth inhibition assays to investigate the drug sensitivity of MDA-MB-453 and CAL-148 cell lines, both categorized as the LAR subtype. Our findings indicate that MDA-MB-453 cells were significantly inhibited by chidamide and showed overall sensitivity to all tested drugs (Table 2). The IC50 values were 1.09 μM, 18.67 μM, 4.12 nM, and 0.77 μM for chidamide, enzalutamide, paclitaxel, and alpelisib, respectively (Fig. 3A, Table 2). CAL-148 cells exhibited increased sensitivity to chidamide, paclitaxel, and alpelisib but demonstrated resistance to enzalutamide, with IC50 values of 2.40 μM, 3.9 nM, 1.09 μM, and 149.3 μM for each drug, respectively (Fig. 3B, Table 2). Interestingly, we demonstrated that both MDA-MB-453 and CAL-148 cells exhibited high sensitivity to chidamide through the mono-drug IC50 assay and confirmed that the non-LAR cell lines exhibited relatively lower sensitivity to chidamide than did the LAR cell lines (Additional file 1: Fig. S1A, B).

**Table 2:** IC50 of a single drug in LAR cell lines

Drugs	MDA-MB-453	CAL-148
Chidamide (μM)	1.09	2.4
Enzalutamide (μM)	18.67	149.3
Paclitaxel (nM)	4.12	3.9
Alpelisib (μM)	0.77	1.09

Then, we investigated the potential synergistic effects of anti-AR therapy in combination with chidamide treatment using drug combination assays and the Chou-Talalay method [32]. A combination index (CI) > 1 indicated an antagonistic effect between the two drugs, while a CI < 1 suggested a synergistic effect. In both MDA-MB-453 and CAL-148 cells, combining low concentrations of chidamide with enzalutamide demonstrated significant synergy, resulting in a 20−50% decrease in growth rate (CI = 0.2, 0.21, respectively) (Fig. 3C). However, the synergistic effects of chidamide in combination with paclitaxel or alpelisib were strongly correlated with drug concentration and cell lines. In MDA-MB-453 cells, synergistic effects were observed only when combining chidamide with high concentrations of paclitaxel (3.7 nM, CI = 0.6) or high concentrations of alpelisib (5.56 μM, CI = 0.6) (Additional file 1: Fig. S1C, D). In CAL-148 cells, chidamide combined with paclitaxel slightly decreased the growth rate, indicating antagonism between the two drugs. Similarly, the combination of chidamide and alpelisib exhibited antagonism, except for the high concentration of alpelisib in CAL-148 cells (Additional file 1: Fig. S1E, F).

Overall, we discovered that chidamide had a more pronounced inhibitory effect on the proliferation of LAR TNBC cell lines than other monotherapies. Furthermore, the combination of chidamide and enzalutamide exhibited a significant synergistic effect.

### Synergistic effect of chidamide combined with enzalutamide in vivo and ex vivo

**Fig. 4:**
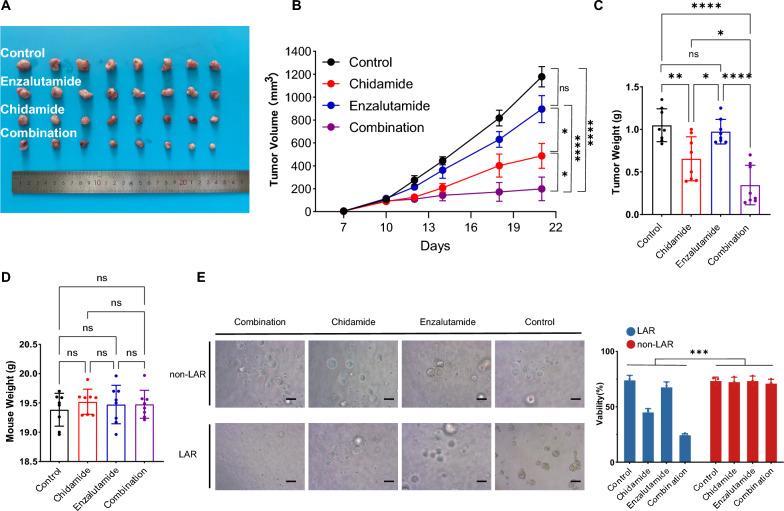
Synergistic effect of chidamide combined with enzalutamide in vivo and ex vivo**. A-D** Representative tumor images **A**, tumor growth curves **B**, endpoint tumor weight **C** bar plots and endpoint mouse weight bar plots (**D**) for different treatment groups. **E** Representative brightfield images of patient-derived organoids (PDOs) on day 5 after drug treatment. PDO models of LAR and non-LAR subtypes were treated with DMSO (control), 1 μM chidamide, 1 μM enzalutamide or the combination therapy for 5 days. Scale bar, 200 μm.Viability was calculated by each group compared with group blank (PDOs without any additional treatment). Abbreviations: LAR, luminal androgen receptor. The data are presented as the mean ± SEM and were compared using Student’s t test (B-D) or Kruskal-Wallis test (E) ****indicates P < 0.0001; ***indicates P < 0.001; **indicates P < 0.01; *indicates P < 0.05; ns indicates no significance

Next, we explored the efficacy and toxicity of chidamide and enzalutamide, both alone and in combination through in vivo experiments. The combination of chidamide and enzalutamide resulted in greater tumor regression in TS/A (mouse LAR tumor cell line) allograft models than treatment with chidamide or enzalutamide alone (Fig. 4A–C). Specifically, we observed a 59% decrease in tumor volumes after treatment with chidamide, a 25% decrease after enzalutamide treatment, and an 84% decrease after the combination treatment. Additionally, we found that tumor weights decreased by 18%, 6%, and 72% following treatment with chidamide, enzalutamide, and the combination, respectively. Importantly, there were no significant differences in body weight loss between the combination group and the groups treated with single agents, indicating the acceptable toxicity of the combination treatment (Fig. 4D). Furthermore, the synergetic effect of chidamide and enzalutamide was confirmed using LAR-subtype patient-derived organoids (PDOs) (Fig. 4E).

In summary, our in vivo experiments demonstrated that the combination of chidamide and enzalutamide led to superior tumor regression. This synergetic effect was further validated ex vivo using LAR-subtype PDOs.

### Potential mechanisms of the synergistic effect of chidamide combined with enzalutamide

**Fig. 5:**
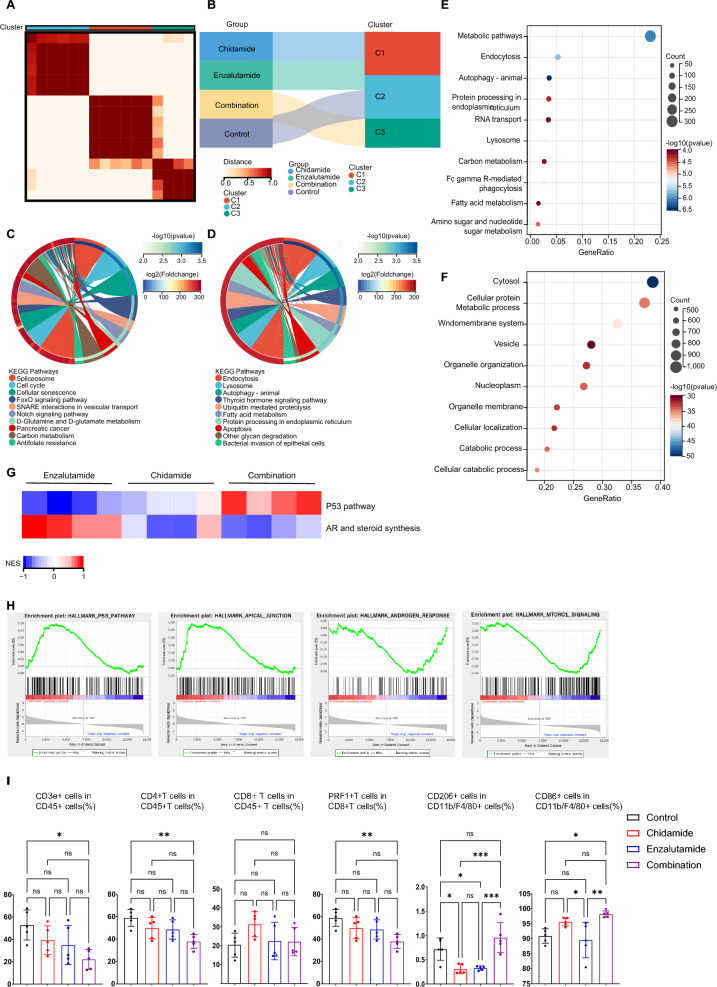
Potential mechanisms of the synergistic effect of chidamide combined with enzalutamide. **A** ConsensusCluster determined by ERGs in TS/A tumor samples. **B** The Sankey diagram displaying relationships among different treatment groups and gene clusters. **C, D** GSVA analysis of enriched pathways in cluster 2 compared with cluster 1 (**C**) and cluster 3 (**D**). **E, F** KEGG (**E**) and GO (**F**) analysis of differentially expressed genes among different treatment groups. **G** GSVA analysis of AR and steroid synthesis, and the P53 pathway among different treatment groups. **H** GSEA analysis of differentially expressed genes among different treatment groups. **I** Bar plots showing the percentage of CD3e^+^ cells among CD45^+^ cells, CD4^+^ cells among CD45^+^ T cells, CD8^+^ cells among CD45^+^ T cells, PRF1^+^ cells among CD3e^+^ CD45^+^ CD8^+^ T cells, CD206^+^ cells among CD45^+^ CD11b^+^ F4/80^+^ cells, and CD86^+^ cells among CD45^+^ CD11b^+^ F4/80^+^ cells. Data were compared using Student’s t test (I) or Mann–Whitney test (C–F)

To elucidate the mechanism associated with the synergistic effect of chidamide and enzalutamide in LAR-subtype TNBC, we conducted RNA sequencing of tumors from the TS/A models. Initially, using consensus clustering, we identified three distinct clusters that exhibited specific associations with different treatment groups (Fig. 5A, B). Specifically, the chidamide group was assigned to cluster 1, while the combination therapy group exclusively fell within cluster 3. This correspondence indicates the presence of distinct mechanisms governing tumor inhibition between the chidamide monotherapy and combination therapy groups. Additionally, resembling the control group, certain samples from the enzalutamide group were affiliated with cluster 2, thereby partially explaining the limited efficacy of enzalutamide treatment. To further elucidate the potential tumor-inhibiting mechanisms of different treatments, we explored the typical biological features of various clusters by GSVA (Gene Set Variation Analysis) enrichment analysis. Cluster 1 was primarily enriched in the cell cycle, cellular senescence and spliceosome, suggesting that these biological processes potentially mediate tumor inhibition led by chidamide monotherapy (Fig. 5C). In comparison to the other two clusters, Cluster 3 exhibited significant enrichment in autophagy, apoptosis and diverse metabolic pathways such as fatty acid metabolism and glyoxylate and dicarboxylate metabolism (Fig. 5D). This suggests that combination therapy may predominantly kill tumor cells by regulating programmed cell death and metabolic pathways. Subsequently, we determined the differentially expressed genes (DEGs) in the combination therapy group compared to the chidamide group and enzalutamide group separately using a cutoff of p value  <  0.05 and log2FC ≥ 1.5. Through KEGG and GO pathway analyses, combination therapy predominantly affects metabolic pathways, as well as autophagy, which aligns consistently with the aforementioned cluster comparison results (Fig. 5E, F).

As previously reported, the LAR subtype is characterized by AR positivity, as well as dysregulated cell-cycle signaling [6]. Such findings provide a rationale for the use of AR antagonists and CDK 4/6 inhibitors as a therapeutic strategy for LAR TNBC. However, this approach did not achieve the expected results [7]. Thus, our investigation aimed to determine whether combination therapy enhances the inhibition of the cell cycle-related pathways and the AR pathway, thereby increasing therapeutic efficacy. Strikingly, the combination therapy group exhibited a significant upregulation of P53 signaling, as well as downregulation of RB and overall cell cycle pathways (Fig. 5G and Additional file 2: Fig. S2A). Likewise, the combination therapy group exhibited distinct patterns compared to the other two monotherapy groups when examining the AR pathway and its downstream signaling through GSVA analysis. Not surprisingly, enzalutamide markedly suppressed AR signaling. In contrast, chidamide monotherapy had a relatively weak impact on AR signaling, while the combination therapy exhibited downregulation of AR and steroid synthesis (Fig. 5G, Additional file 2: Fig. S2A). These results were also confirmed through GSEA (Gene Set Enrichment Analysis) using the above identified DEGs (Fig. 5H). Additionally, GSEA results demonstrated a significant upregulation in the apical junction, along with a downregulation in mTORC1 signaling (Fig. 5H).

Finally, we investigated whether the tumor microenvironment (TME) is involved in the distinct antitumor effects of these treatment strategies. Although the ESTIMATE analysis results demonstrated a lower immune score and stromal score in the combination group, there was no statistically significant difference compared with single drug group (Additional file 2: Fig. S2B). Using flow cytometry and CIBERSORT, we also observed little alteration regarding immune cell composition (Fig. 5I, Additional file 2: Fig. S2C). These findings indicate that the inhibition of tumor proliferation by combination therapy may not mainly rely on the immune response.

In conclusion, the combination of chidamide and enzalutamide primarily inhibits tumor proliferation by regulating metabolism, particularly fatty acid metabolism and autophagy. Moreover, the effectiveness of combination therapy, which exhibits a more pronounced effect than solely antagonizing AR, may be attributed to the suppression of the steroid synthesis and cell cycle-related pathways. Additionally, the antitumor tumor microenvironment appears to have limited impacts on the synergistic effect of the combination therapy.

## Discussion

The LAR subtype in TNBC presents a formidable challenge, as seen in the low pathologic complete response rates postneoadjuvant chemotherapy and limited objective response rates in metastatic TNBC after AR inhibitor therapy. This study aims to explore promising treatment approaches for LAR-subtype TNBC. Employing the extensive FUSCC dataset, we conducted a multiomics analysis and revealed HDACs as potential promising targets for the LAR-subtype TNBC. Through in vitro, in vivo and ex vivo experiments, we examined and confirmed the significant antitumor effect of chidamide in LAR-subtype cases. This effect can be further enhanced when in combination with enzalutamide. Moreover, our findings indicate that chidamide combined with enzalutamide does not significantly increase toxicity, further supporting the potential of this combination therapy for LAR TNBC patients. Additionally, transcriptome sequencing shed light on the mechanisms underlying this synergy, implicating autophagy, apoptosis, fatty acid metabolism, and glyoxylate and dicarboxylate metabolism.

This study has potential for clinical translation. The FUTURE clinical trial demonstrated the limited effect of anti-AR and anti-CDK4/6 therapies for LAR patients, which emphasized the necessity of investigating alternative treatment strategies [7]. Of note, our study proposed and confirmed that the combination therapy of chidamide and enzalutamide may be a promising therapeutic strategy for LAR-subtype TNBC. Crucially, the safety of such combination therapy was acceptable. Additionally, our work provides a theoretical basis for clinical implementation. In a previous study, chidamide treatments were found to stimulate cell apoptosis by promoting ULK2-mediated autophagy and increasing sensitivity to doxorubicin in breast cancer [33]. Moreover, the combination of chidamide and doxorubicin can induce p53-driven cell cycle arrest and cell apoptosis, as well as reverse multidrug resistance in breast cancer [34]. Consistent with these findings, our study revealed upregulation of autophagy and P53 signaling in the combination group. Additionally, chidamide could elicit immunogenic cell death within TNBC, enhancing cancer immunogenicity and promoting an antitumor immune response [35]. However, it is important to note that our study’s observations regarding the tumor microenvironment and immune signatures indicate that the combination of chidamide and enzalutamide may not significantly enhance the antitumor immune response.

Several limitations should be acknowledged. First, this study lacks in vivo evidence to support the efficacy of treatment strategies in patient populations. However, we have verified its effectiveness in multiple models, including in vitro cell lines, in vivo allograft tumor models and even ex vivo patient-derived organoid models. Thus, our study provides relatively strong evidence for clinical translation. Second, this study identified potential mechanisms through transcriptomic data analysis, but experimental evidence is lacking. Notably, the primary objective of this study was to discover novel therapeutic strategies for LAR patients. Additionally, our findings indicate that combination therapy has the potential to influence autophagy and metabolic pathways in tumors. The regulatory mechanisms related to this phenomenon have been previously reported and confirmed [33–35].

## Conclusion

Overall, this study sheds light on the importance of considering the distinct characteristics and epigenetic regulation of the LAR subtype in the development of targeted therapies. This finding underscores the potential of chidamide and enzalutamide combination therapy as a promising treatment option for LAR-subtype TNBC.

### Supplementary Information

**Additional file 1: Figure S1.** Drug combination assays for chidamide and paclitaxel or alpelisib in LAR subtype cell lines. **A, B** IC50 test of chidamide in non-LAR cell lines Hs 578T (**A**) and HCC1806 (**B**). **C, D** Heatmap showing the cell viability of chidamide and alpelisib (**C**) or paclitaxel (**D**) in MDA-MB-453 at different drug concentrations. **E, F** Heatmap showing the cell viability and CI value of chidamide and alpelisib (**E**) or paclitaxel (**F**) in CAL-148 at different drug concentrations.* indicates CI>2

**Additional file 2: Figure S2.** Potential mechanisms of the synergistic effect of chidamide combined with enzalutamide. **A** GSVA analysis of the RB pathway, cell cycle, and AR signaling among different treatment groups. **B** Box plots showing the ImmuneScore and StromalScore calculated by ESTIMATE. **C** Bar plots depicting the immune signatures based on CIBERSORT analysis. Statistical analysis was performed by the Mann–Whitney test. ****indicates P < 0.0001; ***indicates P < 0.001; **indicates P < 0.01; *indicates P < 0.05; ns indicates no significance.

## Data Availability

All data analyzed during this study are included in this paper and/or the Supplemental Materials or are available on reasonable request via the correspondence. The clinical data and omics data can be viewed in The National Omics Data Encyclopedia (NODE) (http://www.biosino.org/node) by pasting the accession (OEP000155) into the text search box or through the URL: http://www.biosino.org/node/project/detail/OEP000155.

## Abbreviations

TNBC: Triple-negative breast cancer
LAR: Luminal androgen receptor
HDACs: Histone deacetylases
AR: Androgen receptor
ER: Estrogen receptor
PR: Progesterone receptor
HER2: Human epidermal growth factor receptor 2
FDA: Food and Drug Administration
NMPA: National Medical Products Administration
FUSCC: Fudan University Shanghai Cancer Center
ATCC: American Type Culture Collection
CI: Combination index
ESTIMATE: Estimation of STromal and Immune cells in MAlignant Tumor tissues using Expression data
WES: Whole-exome sequencing
SCNA: Somatic copynumber alteration
PD: Progressive disease
SD: Stable disease
ERGs: Epigenetic-related genes
GO: Gene Ontology
KEGG: Kyoto Encyclopedia of Genes and Genomes
WGCNA: Weighted gene coexpression network analysis
PDOs: Patient-derived organoids
GSVA: Gene set variation analysis
DEGs: Differentially expressed genes
GSEA: Gene set enrichment analysis
TME: Tumor microenvironment
PPI: Protein–protein interaction
