# Supplementary material for: Enhancing therapeutic efficacy in luminal androgen receptor triple-negative breast cancer: exploring chidamide and enzalutamide as a promising combination strategy
Source: Cancer Cell Int. 2024 Apr 9;24:131. doi: 10.1186/s12935-024-03313-5 (PMC11003165; doi:10.1186/s12935-024-03313-5)
Supplement: Supplementary file 1 — Additional file 1: Figure S1. Drug combination assays for chidamide and paclitaxel or alpelisib in LAR subtype cell lines. A, B IC50 test of chidamide in non-LAR cell lines Hs 578T (A) and HCC1806 (B). C, D Heatmap showing the cell viability of chidamide and alpelisib (C) or paclitaxel (D) in MDA-MB-453 at different drug concentrations. E, F Heatmap showing the cell viability and CI value of chidamide and alpelisib (E) or paclitaxel (F) in CAL-148 at different drug concentrations.* indicates CI>2 [file 12935_2024_3313_MOESM1_ESM.pdf]

Figure S1

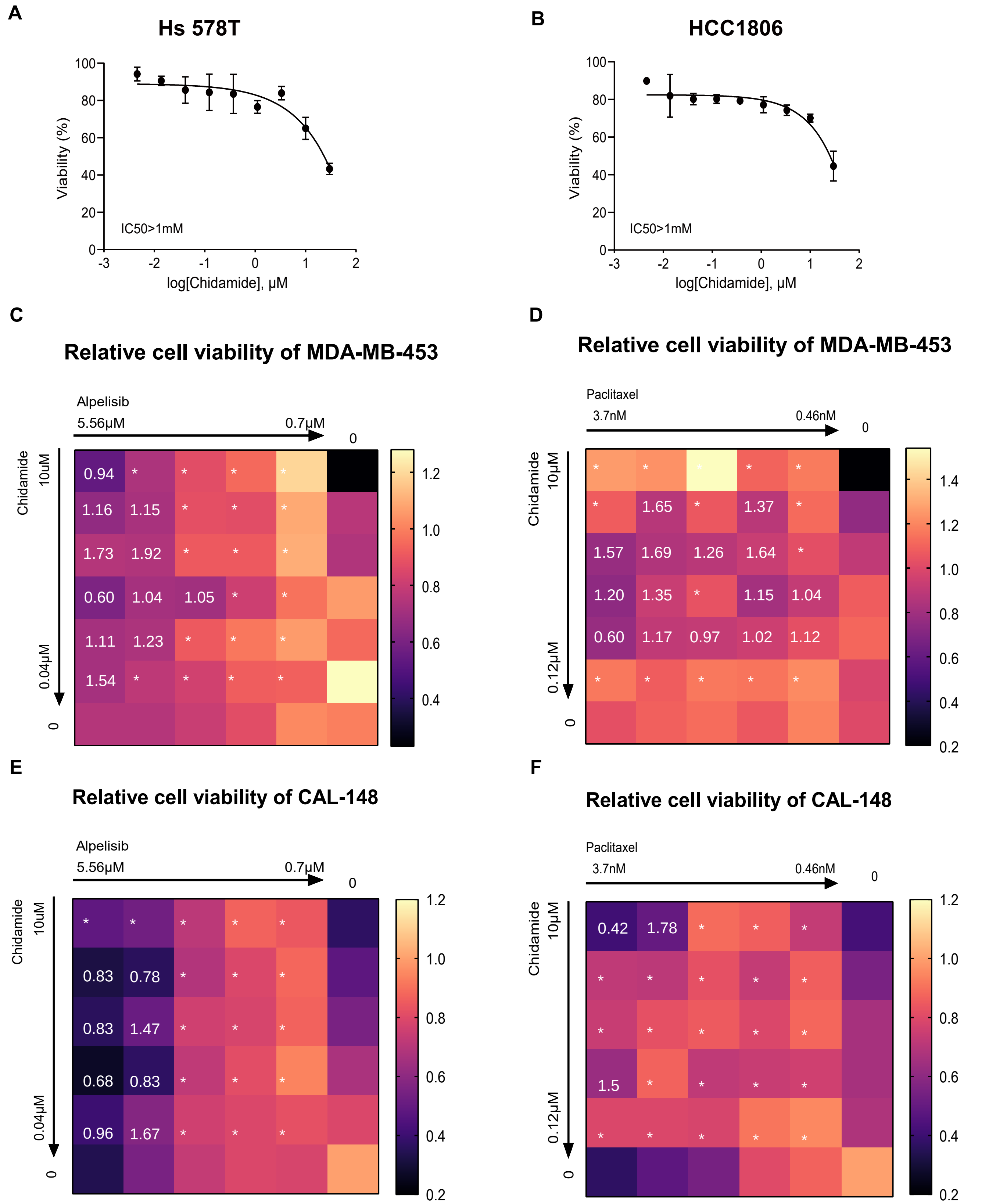

Figure S1 Drug combination assays for chidamide and paclitaxel or alpelisib in LAR subtype cell lines **(A-B)** IC50 test of chidamide in non-LAR cell lines Hs 578T (A) and HCC1806 (B). **(C-D)** Heatmap showing the CI value of chidamide and alpelisib (C) or paclitaxel (D) in MDA-MB-453 at different drug concentrations. **(E-F)** Heatmap showing the CI value of chidamide and alpelisib (E) or paclitaxel (F) in CAL-148 at different drug concentrations.* indicates CI>2
