# Supplementary material for: Enhancing therapeutic efficacy in luminal androgen receptor triple-negative breast cancer: exploring chidamide and enzalutamide as a promising combination strategy
Source: Cancer Cell Int. 2024 Apr 9;24:131. doi: 10.1186/s12935-024-03313-5 (PMC11003165; doi:10.1186/s12935-024-03313-5)
Supplement: Supplementary file 2 — Additional file 2: Figure S2. Potential mechanisms of the synergistic effect of chidamide combined with enzalutamide. A GSVA analysis of the RB pathway, cell cycle, and AR signaling among different treatment groups. B Box plots showing the ImmuneScore and StromalScore calculated by ESTIMATE. C Bar plots depicting the immune signatures based on CIBERSORT analysis. Statistical analysis was performed by the Mann–Whitney test. ****indicates P < 0.0001; ***indicates P < 0.001; **indicates P < 0.01; *indicates P < 0.05; ns indicates no significance. [file 12935_2024_3313_MOESM2_ESM.pdf]

## Figure S2

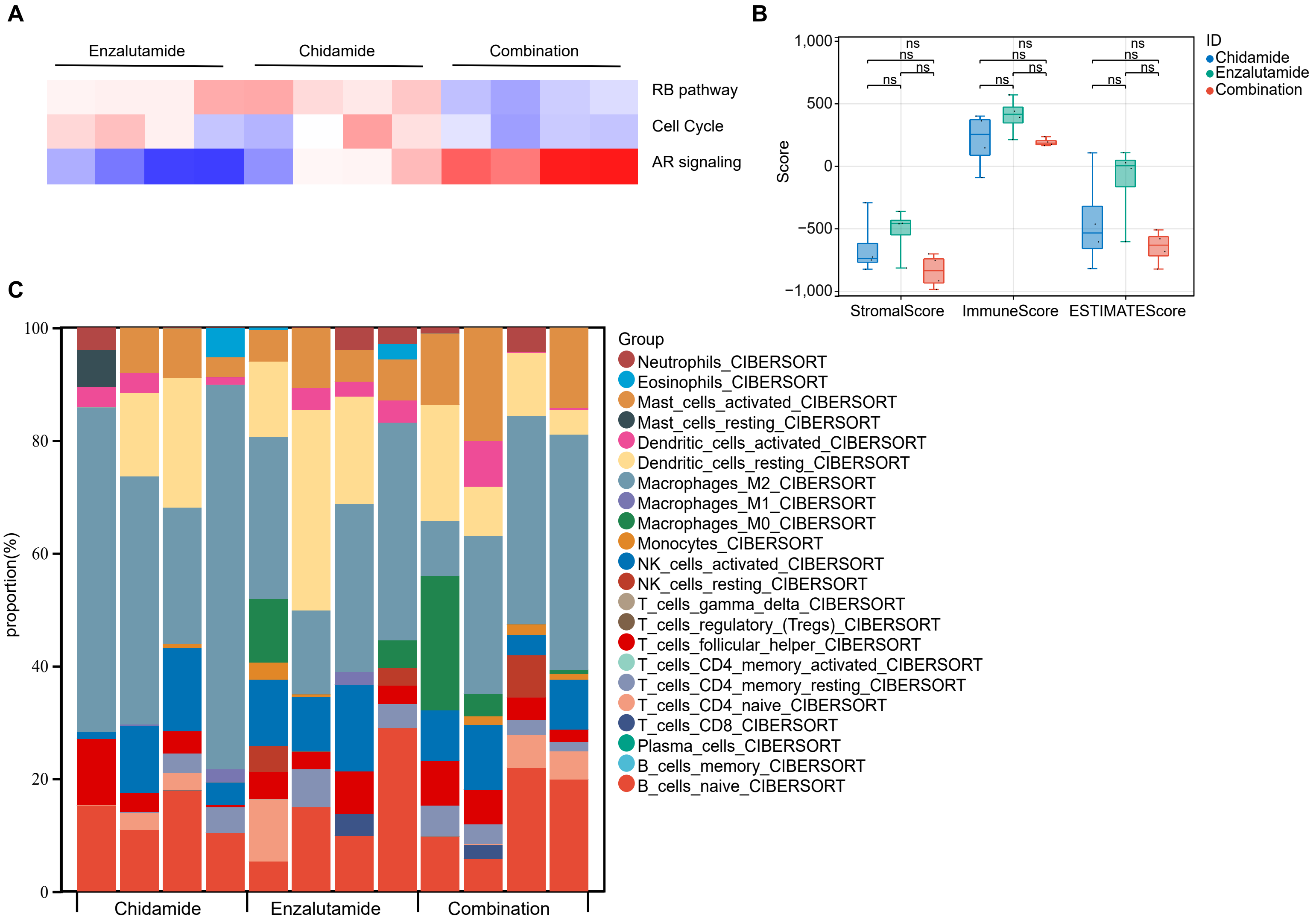


**Figure S2** Potential mechanisms of the synergistic effect of chidamide combined with enzalutamide

**(A)** GSVA analysis of the RB pathway, cell cycle, and AR signaling among different treatment groups.

**(B)** Box plots showing the ImmuneScore and StromalScore calculated by ESTIMATE.

**(C)** Bar plots depicting the immune signatures based on CIBERSORT analysis.

Statistical analysis was performed by the Mann–Whitney test. **** indicates $P < 0.0001$; *** indicates $P < 0.001$; ** indicates $P < 0.01$; * indicates $P < 0.05$; ns indicates no significance.
